# Isolated cryptococcosis of a lumbar vertebra in an immunocompetent patient: A case report and literature review

**DOI:** 10.3389/fsurg.2022.1079732

**Published:** 2023-01-06

**Authors:** Zhongxiong Jia, Min Tang, Xiaojun Zhang, Xiaojuan Xin, Wei Jiang, Jie Hao

**Affiliations:** ^1^ Department of Orthopedics, The Second People's Hospital of Yibin, Yibin, China; ^2^ Department of Oncology, The First Affiliated Hospital of Chongqing Medical University, Chongqing, China; ^3^ Department of Orthopedics, The First Affiliated Hospital of Chongqing Medical University, Chongqing, China; ^4^ Infectious Disease Department, The First Affiliated Hospital of Chongqing Medical University, Chongqing, China

**Keywords:** *Cryptococcus*, spinal infection, lumbar vertebral cryptococcosis, case report, surgery

## Abstract

**Background:**

*Cryptococcus*, a kind of fungus, can be found in soil, decayed wood, and avian excreta. Immunocompromised patients are prone to infection caused by *Cryptococcus*, and the lungs and central nervous system are the main target organs. Cryptococcosis rarely occurs in the lumbar vertebra or in immunocompetent patients.

**Case presentation:**

A 40-year-old adult male with isolated lumbar vertebra cryptococcosis at the L4 vertebra underwent successful lesion removal surgery performed *via* the posterior approach and postoperative administration of an antifungal agent. At the 12-month follow-up, the patient's pain was relieved, and his motor function had improved. Isolated *Cryptococcus* vertebrae infection is a rare infectious disease.

**Conclusions:**

A needle biopsy can confirm the diagnosis of *Cryptococcus* infection. When patients present with unbearable symptoms of nerve compression, posterior depuration combined with postoperative antifungal agents is a good option.

## Introduction

*Cryptococcus* is a fungus similar to yeast that lives in bird droppings, decaying wood, and soil (1). The respiratory tract is the main route of transmission, and the susceptible population includes people with low immune function. Ninety percent of the cases occur in acquired immune deficiency syndrome (AIDS) patients, which can involve multiple organs throughout the body but mainly involves the central nervous system and lungs (2–5). Skeletal infection caused by *Cryptococcus* is relatively rare, accounting for approximately 5% of all cases of *Cryptococcus* infection (6), and the common sites are the lumbar spine, pelvis, ribs, and skull (7). To the best of our knowledge, only a few studies have reported spinal infections caused by *Cryptococcus*. We report a case of cryptococcosis of the lumbar vertebra in an immunocompetent patient with complete clinical data to raise surgeons’ awareness of cryptococcosis of the spine.

## Case report

**Figure 1:**
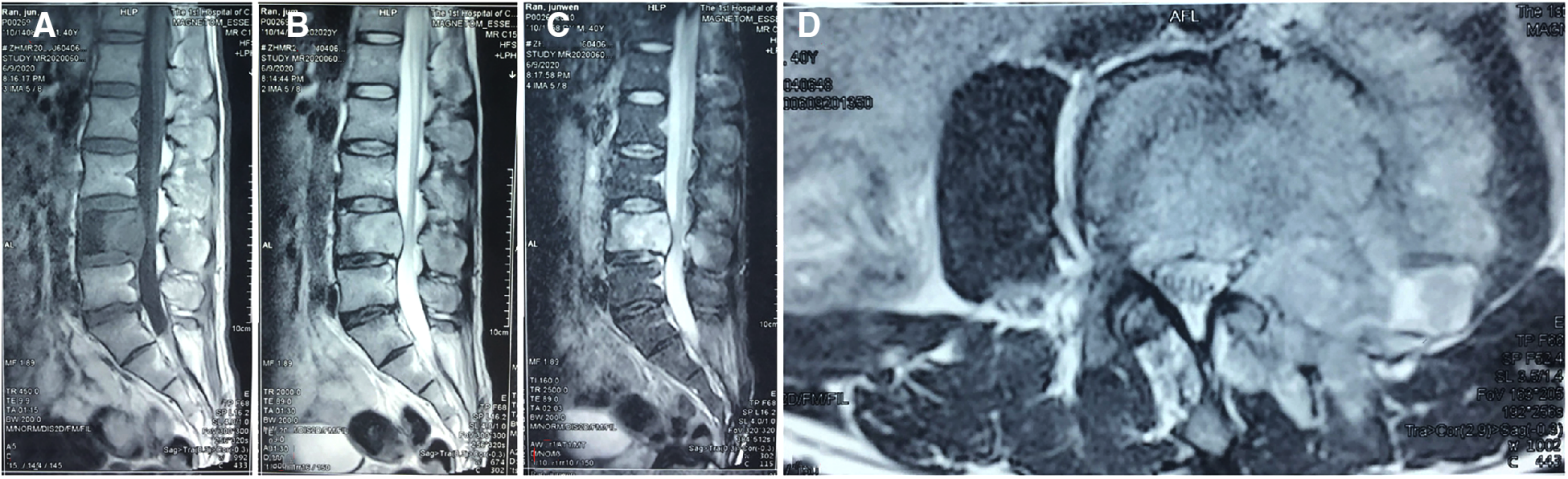
MRI scan showing the paravertebral soft tissue mass and the spinal canal stenosis and the pedicle of the fourth lumbar vertebra. (**A**) T1-weighted images, (**B**) T2-weighted images, (**C**) short tau inversion recovery, and (**D**) transverse section imaging.

A 40-year-old adult male labourer, who was a construction worker mainly engaged in the handling of construction materials, presented with a more than 4-month history of low back pain, pain radiating to the left limb (visual analogue scale score of 9; Oswestry Disability Index score of 70%), and left limb numbness, without symptoms of tuberculosis, such as fever, night sweats, or cough. A physical examination revealed weakness of the left limb of approximately grade IV, sensory disturbance in the left L4 and L5 area, and difficulty in stretching the left hip. The bilateral Achilles tendon and knee jerk reflexes were normal. There was localised tenderness in the lower lumbar spine. The patient had no medical history of tuberculosis, tumour, AIDS, operations, sarcoidosis, treatment with corticosteroids, or organ transplantation. His close relatives had no history of cancer, tuberculosis, *Cryptococcus*, or other diseases. He denied a past exposure to bird droppings or decaying wood. Therefore, we did not find the source of infection. Lumbar x-ray was performed, which showed that the left pedicle of L4 was unclear and was suspected to be bone destruction. Computed tomography (CT) revealed a lytic lesion at the L4 vertebrae. The entire left half of the vertebral body was involved. The left side of the L4 vertebral body was obviously damaged, and the lesion involved the paravertebral soft tissue. A single photon emission computed tomography (SPECT) scan showed increased uptake in the L4 vertebrae. SPECT did not find any further lesions except the L4 vertebra. Magnetic resonance imaging (MRI) revealed bone destruction in the L4 vertebral body and a portion of the spinal column enclosure. Sagittal T1-weighted MRI of the lumbar spine demonstrated areas of diffuse low signal intensity in L4. Sagittal T2-weighted MRI of the lumbar spine showed a high-intensity zone of oedema around the areas of isointensity in L4. The endplates of the L4 vertebral body were involved, and the upper and lower discs of the L4 vertebra were normal. A transverse MRI scan showed a paraspinal soft tissue lesion that looked like a tumour in L4 (Figure 1). Laboratory investigations revealed that the erythrocyte sedimentation rate (ESR) was 57 mm/h. C-reactive protein (CRP) and procalcitonin were normal. Blood counts, liver and renal function, and other serum chemistries were also normal. The enzyme linked immunosorbent assay (ELISA) test for AIDS was negative. We performed a needle biopsy surgery to identify the nature of the lesion. The pathologist found *Cryptococcus* in the lesion; thus, the pathological examination suggested cryptococcal infection. After needle biopsy surgery, we drew a sample of the patient's blood for cryptococcal antigen detection, which was positive. At the same time, the patient was examined by chest CT and brain MRI, and no abnormality was found. We suggested surgical treatment for the patient, but he was concerned about the risk of surgery, refused the operation, and required conservative treatment. The patient was referred to the infection department for antifungal therapy. However, in the course of antifungal treatment with oral fluconazole (400 mg/day) for approximately 2 weeks, the lower limb pain symptoms continued to worsen, so the patient returned to our department for surgical treatment. We performed a posterior approach surgery to remove the lesion and relieve spinal nerve compression.

This study was performed according to the guidelines of the Declaration of Helsinki and its amendments. Written informed consent was obtained from the patient for the publication of this study and any accompanying images.

**Figure 2:**
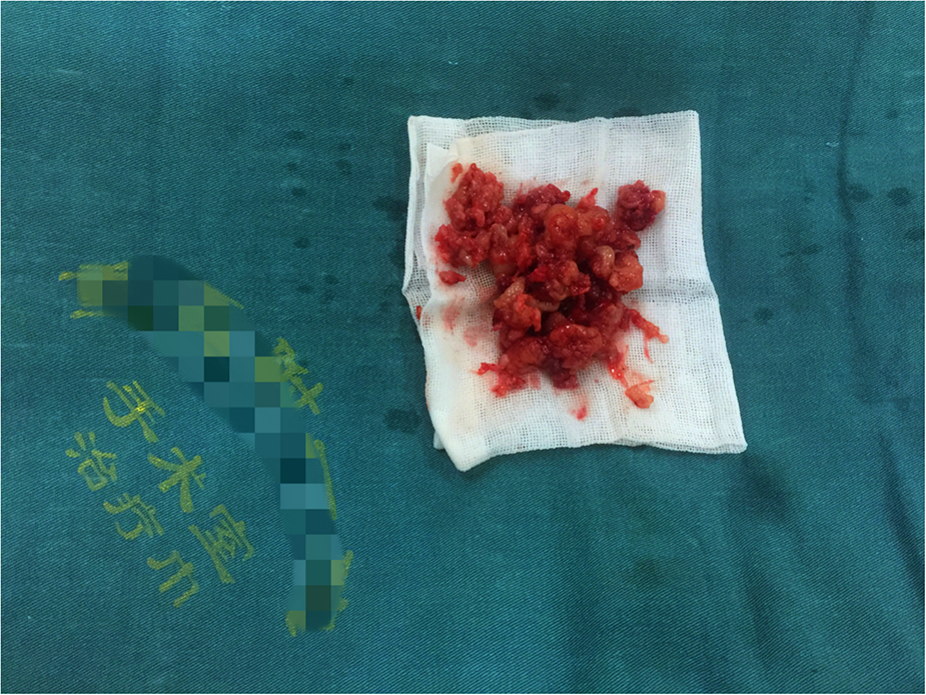
The lesions look like jelly.

**Figure 3:**
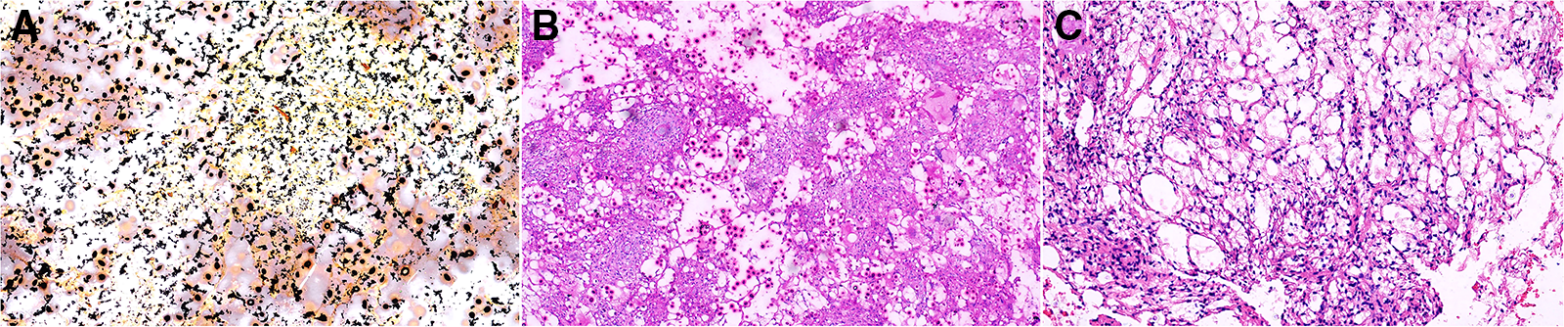
Pathological: (**A**) Ag (×100), (**B**) PAS (×100), and (**C**): HE (×100). PAS, Periodic Acid-Schiff stain; HE, hematoxylin-eosin staining.

Under general endotracheal anaesthesia, the patient was placed on the operating table in a prone position. At the affected section of the spine, a standard posterior middle approach was made. Through lateral subperiosteal dissection, the resected levels were exposed to the facet joints in the lumbar region. Pedicle screws were inserted one level above and below the lesion by the freehand technique. When inserting pedicle screws into the L4 vertebra, we found that the accessory structure of the left vertebral body had been destroyed so that pedicle screws could not be placed. Therefore, pedicle screws were not placed on the left side of the L4 vertebral body. After all pedicle screws had been inserted into the centre of the pedicles, the laminae, articular processes, and spinous processes at the level of the lesions were resected. The dura and L4 and L5 nerve roots were then carefully exposed. Then, the lesions were debrided by bone curettes and pituitary rongeurs. The lesions looked like jelly (Figure 2). Simultaneously, 360° decompression around the canal and roots was completed. We filled the lesion with a fluconazole-soaked gelatine sponge to provide local antifungal therapy and used longitudinal beams to connect with the pedicle screws to build a complete internal fixture. The resected lesions were histopathologically examined (Figure 3).

**Figure 4:**
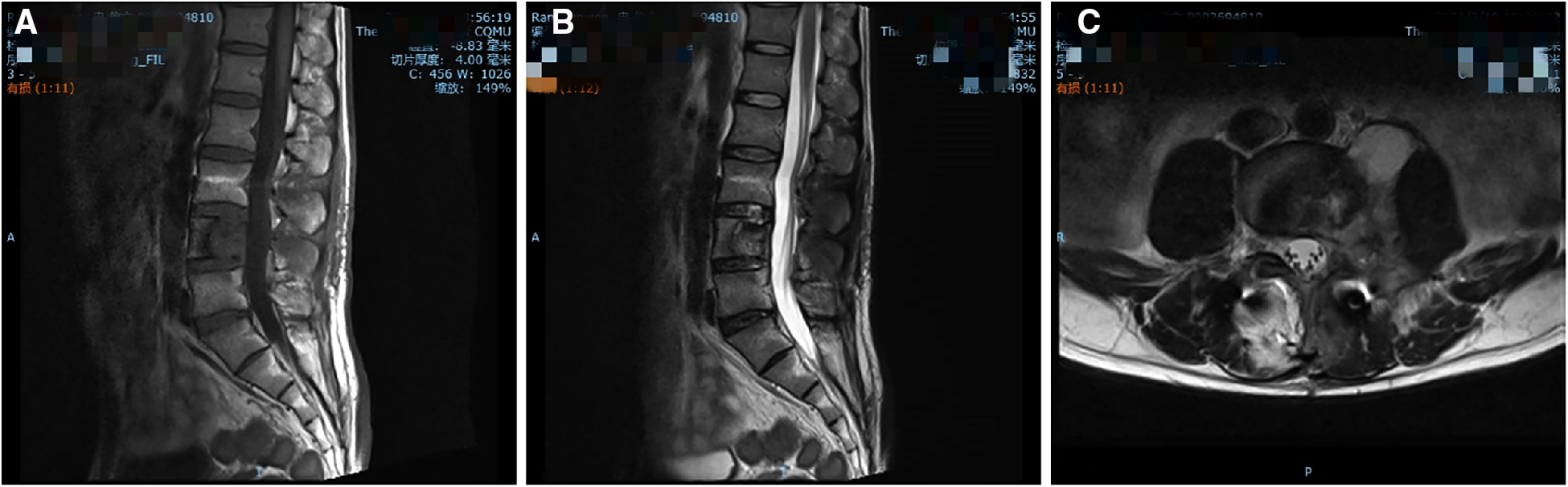
Twelve-month follow-up MRI shows a significant reduction of the lesion. (**A**) T1-weighted, (**B**) T2-weighted, and (**C**) axial images.

Mannitol (125 ml/day) and dexamethasone (10 mg/day) were administered intravenously for 3 days following surgery to relieve nerve root oedema and inflammation. The patient was administered oral fluconazole (400 mg/day) as an antifungal treatment. The patient left the hospital approximately 1 week after surgery. Three months following the spinal surgery, the patient reported relief of his symptoms and had returned to his normal preoperative activities. Physical examination revealed that the left limb strength, sensation in his left L4 and L5 areas, and left hip activity had returned to normal. His erythrocyte sedimentation rate was 41 mm/h, which is higher than normal. Twelve months postoperatively, follow-up MRI images of the lumbar spine showed a significant reduction of the lesion (Figure 4).

## Discussion

Skeleton infection caused by *Cryptococcus* is relatively rare, accounting for approximately 5% of all cases of *Cryptococcus* infection (6). However, cryptococcal spine infections are the most common site of bone infection by *Cryptococcus* (8). We performed comprehensive research *via* PubMed on cryptococcal spine infections, which were reported in a total of 17 articles (Table 1). Unfortunately, the full text of three of the articles could not be found. Upon reviewing the 14 published studies, we found 14 cases (9–22). The clinical features of cryptococcal spine lesions were atypical. Fever, cough, pain at the infected site, and radiating pain were the most common symptoms. Incontinence of urine and faeces and full paraplegia occurred in some severe cases (10, 13, 14). The above symptoms are similar to those of spinal tumours and spinal tuberculosis. In our case, the patient presented with low back pain and pain radiating to the left limb. The patient had difficulty straightening the left hip and continually flexed the left lower limb. Paravertebral lesions were considered to have invaded the iliopsoas muscle. During antifungal treatment in the infection department, the patient's lower limb pain symptoms continued to worsen. Surgery was performed, and the patient fully recovered after 1 year of follow-up.

**Table 1:** General characteristics of 14 literature studies with spine cryptococcosis.

Study	Age (year)/Sex	Site	Symptoms	CAT	ESR (mm/h)	CRP (mg/L)	Therapy	Medical history	Outcome
Singh and Xess (20)	29/F	L5	Fever, cough, backache, swelling over the sternum	P	110*	NA	Amphotericin B, operation	First-trimester spontaneous abortion	Died
Wang et al. (21)	67/F	T2–T3	Backache and occasional pain radiating bilaterally to the shoulders and chest	P	80*	24.43*	Surgery, voriconazole	No	Curative
Minta (9)	26/M	L4–L5	Backache and pain radiating to lower limbs	NA	NA	NA	Fluconazole, tenofovir–vudine–nevirapine	AIDS	Improve
Al-Tawfiq and Ghandour (11)	34/F	L4	Intermittent fever, low back pain radiating to the right lower	N	89*	8.2*	Nafcillin, flucoconazole, isoniazid, rifampin, ethambutol, pyrazinamide	Pulmonary tuberculosis	Curative
Gurevitz et al. (15)	67/F	L3	Low back pain, fever	P	70*	NA	Biopsy, amphotericin B, 5-fluorocytosine	Lymphadenopathy	Curative
Govender and Charles (13)	9/F	T4	Backache weakness of the lower limbs incontinent of faeces and urine	NA	82*	NA	Surgery, amphotericin B, flucytosine	Pulmonary tuberculosis	Died
Glynn et al. (12)	52/F	L2	Low back pain	P	20*	NA	Biopsy, amphotericin B, 5-flucytosine, ketoconazole	No	Improve
Gupta et al. (14)	24/F	T2–T3	Pain in left shoulder and left chest, paresthesias in both lower limbs, paraparesis and urinary incontinence	NA	NA	NA	Surgery, anti-tubercular therapy, amphotericin B, flucytosine	Tuberculous cervical lymphadenopathy	Died
Jain et al. (16)	72/F	T6	Fever, cough, acute girdle pain at T6 and difficulty in walking	NA	70*	NA	Anti-tubercular therapy, amphotericin B, flucytosine	Diabetic	Improve
Zhou et al. (22)	40/F	L4	Low back pain that had been radiating to the left leg	N	22*	1.45*	Biopsy, fluconazole	Rheumatoid arthritis, scleroderma	Curative
Joo et al. (17)	66/F	L1–L2	Back pain	N	28*	1	Surgery, amphotericin B, fluconazole	Rectal cancer	Curative
Legarth et al. (18)	59/M	T6–T7, 10th rib	Fever, weight loss, a non-fluctuant and tender mass related to the left posterolateral 10th rib	NA	NA	130*	Surgery, voriconazole, fluconazole, itraconazole	NO	Improve
Li et al. (19)	17/F	L1	Fever, back pain, night sweats, headache, nausea	P	NA	NA	Anti-tubercular therapy, hydrocortisone sodium, 5-flucytosine fluconazole, surgery	No	Curative
Adsul et al. (10)	45/F	T4	Back pain, fully paraplegic	NA	38*	18*	Biopsy, surgery, amphotericin B, flucytosine	Type II diabetes	Improve

ESR, erythrocyte sedimentation rate; CRP, C-reactive protein; CAT, cryptococcal antigen test; F, female; M, male; P, positive; N, negative; NA, not available.

*Unnormal.

Imaging examinations are essential for the diagnosis of cryptococcal infection of the spine. Plain x-rays can present difficulty in finding lesions (11, 20, 21), as in our case. However, in the case of Joo et al., plain radiographs showed multiple sclerotic lesions (17). Plain radiographs may show scoliosis in patients with tuberculosis of the spine (19). CT may be a good imaging method for the diagnosis of cryptococcal infection of the spine, as it can show osteolytic lesions in the vertebral body (12, 14–19, 21). In our case, the SPECT scan showed increased uptake in the L4 vertebra. This finding is consistent with that of Zhou et al. and Al-Tawfiq and Ghandour (11, 22). MRI may be a good approach to distinguish between cryptococcal infection of the spine, tumours, and tuberculosis. MRI of the spine always presents a paraspinal soft tissue lesion with vertebral erosion at the level of the infection site and intact disc space above and below the lesion (10, 13, 14, 21, 22). Spinal tuberculosis can destroy the disc space above and below the lesion by approximately 70%, while cryptococcosis of the spine does not (14, 23). It is difficult to distinguish vertebral tumours and cryptococcosis of the vertebrae with a simple imaging examination. Needle biopsy may be a good method for resolving the diagnosis. In our case, we highly suspected that the disease was a spinal tumour when the patient first arrived at our outpatient department. The result of the biopsy showed the finding of *Cryptococcus* in the lesion tissues. ESR, CRP, and cryptococcal antigen test (CAT) can be used as primary screening methods. After reviewing the literature, we found that the ESR was abnormal in 10 cases, CRP was abnormal in 4 cases, and CAT was false negative. The accuracy was approximately 66% in immunocompetent patients with cryptococcosis (24). CAT tests were performed in eight cases, among which five were positive and three were negative (Table 1). In our case, the CAT test was positive, and ESR and CRP increased.

Antifungal therapy plays an important role in the treatment of spinal infections caused by *Cryptococcus*. We should pay attention to the side effects of antifungal drugs during antifungal therapy. In the case presented by Legarth et al., the patient experienced continuous photosensitivity and pruritus during voriconazole treatment. The complication disappeared after the treatment was changed to fluconazole (18). In our case, the antifungal treatment was oral fluconazole (400 mg daily) until 6 months after surgery. No side effects occurred during the treatment. Oral fluconazole (400 mg daily) may be a good choice for treating spinal infections caused by *Cryptococcus*.

In conclusion, there is no standard therapy regimen to treat cryptococcosis of the spine. We recommend surgery as early as possible when the patient's radiating pain in the lower limbs continues to worsen, combined with antifungal drugs after the operation. This treatment plan can quickly enhance a patient's recovery.

## Data Availability

The original contributions presented in the study are included in the article/Supplementary Material, further inquiries can be directed to the corresponding author.
